# Inbred rat heredity and sex affect oral oxycodone self-administration and augmented intake in long sessions: correlations with anxiety and novelty-seeking

**DOI:** 10.1371/journal.pone.0314777

**Published:** 2025-03-10

**Authors:** Burt M. Sharp, Shuangying Leng, Jun Huang, Caroline Jones, Robert W. Williams, Hao Chen

**Affiliations:** 1 Department of Genetics, Genomics and Informatics, College of Medicine, University of Tennessee Health Science Center, Memphis, Tennessee, United States; 2 Department of Pharmacology, Addiction Science and Toxicology, University of Tennessee Health Science Center, Memphis, Tennessee, United States; Technion Israel Institute of Technology, ISRAEL

## Abstract

Oxycodone abuse frequently begins with prescription oral oxycodone, yet vulnerability factors (e.g. sex, genetics) determining abuse are largely undefined. We evaluated genetic vulnerability in a rat model of oral oxycodone self-administration (SA): increasing oxycodone concentration/session (0.025-0.1mg/ml; 1-, 4-, and 16-h) followed by extinction and reinstatement. Active licks and oxycodone intake were greater in females than males during 4-h and 16-h sessions (p < 0.001). Both sexes increased intake between 4-h and 16-h sessions (p < 2e-16), but a subset of strains augmented intake at 16-h (p = 0.0005). Heritability (*h*^2^) of active licks during 4-h sessions at increasing oxycodone dose ranged from 0.30 to 0.53. Under a progressive ratio (PR) schedule, breakpoints were strain-dependent (p < 2e-16). Cued reinstatement was greater in females (p < 0.001). Naive rats were assessed using elevated plus maze (EPM), open field (OF), and novel object interaction (NOI) tests. We correlated these behaviors with 28 parameters of oxycodone SA. Anxiety-defining EPM traits were most associated with SA in both sexes, whereas OF and NOI traits were more associated with SA in males. Sex and heredity are major determinants of motivation to take and seek oxycodone; intake augments dramatically during extended access in specific strains; and anxiety correlates with multiple SA parameters across strains.

## Introduction

Opioids such as morphine and oxycodone have long been prescribed for effective analgesia. However, the prevalence of prescriptions for opioids such as oxycodone, has led to widespread abuse and deaths. Approximately 12 million people misused opioids in 2016 [1]. By 2017, deaths from opioid overdoses rose to approximately 12 million, and in 2022, 80,000 opioid-involved overdose deaths were reported [2].

Most commonly, individuals initiate their habitual intake of oxycodone with prescription oral oxycodone [3,4]. Since pharmacokinetic parameters are important determinants of abuse potential, we designed a rat oral operant self-administration (SA) model. Rats were used to model the oral pattern of drug intake common in humans. Previous reports of oral oxycodone SA [5–7] required training procedures to initiate drug SA that alter the motivation to obtain drugs. The model we developed [8] utilizes operant licking for oxycodone and does not require water restriction or prior drug exposure.

The transition from deliberately controlled drug intake to compulsive drug seeking and taking, characteristic of addiction [9], is often accompanied by a sharp rise in drug use. The marked variation in amount of drug consumed and the extent of addictive behavior observed among individuals depends on multiple factors. In human twin studies, approximately half of the variance in vulnerability to develop an opiate use disorder (OUD) is heritable [10,11]. Complex, sex-dependent patterns also modulate opiate use. In general, male rats self-administer more oxycodone than females at early stages, while females are more susceptible to opioid reward [12].

Based on the known impact of genetics and sex on the vulnerability to OUD, the purpose of these studies was to identify the genetically determined behavioral parameters of oral oxycodone self-administration. To accomplish this, we studied the patterns of drug intake in both sexes during 4-h and 16-h extended sessions; a total of 36 fully inbred rat strains were evaluated, 23 matched by sex. These strains are part of the Hybrid Rat Diversity Panel (HRDP), a set of diverse inbred strains and their isogenic F1 progeny with fully sequenced genomes [13,14]. All inter-sex statistical comparisons were restricted to the 23 sex-matched strains. We also correlated oxycodone SA parameters to exploratory behaviors and locomotion in drug naive rats.

Some strains manifest greater than a 100-fold *increase in oxycodone intake* during our 65-day protocol. We also found significant sex and strain differences that affect multiple parameters of oxycodone SA, including the dramatic strain-specific augmentation of intake with increased drug availability during 16-h access sessions and the correlation of intake between 4- vs 16-h sessions in both sexes. We confirmed the significant heritability (*h*^*2*^) of multiple parameters of oxycodone SA. These findings are similar to those reported in seven inbred rat strains [8] and in studies of human twins [11]. Overall, this oral model of oxycodone SA captures multiple behavioral parameters involved in the strong human abuse potential of oral oxycodone. Drug pharmacokinetics plays an important role in their abuse liability [15,16]. Although the pharmacokinetic properties of oxycodone are quite different in rat vs human [17,18], both species load up when drug is available, develop oxycodone dependency that shows substantial heritability and individual variation, and exhibit sex-dependent differences in consumption patterns. Hence, this rat model will likely have considerable translational potential because the genetic determinants of various oxy-dependent behaviors probably exhibit substantial overlap between the two species.

## Methods

### Animals

Breeders from the HRDP were obtained from Dr. Melinda R. Dwinell at the Medical College of Wisconsin. All rats were bred on campus and housed in groups in a room with a 12:12 h reversed light cycle (lights off: 9AM-9PM) at the University of Tennessee Health Science Center. Experiments were conducted during the dark phase of this cycle. Each rat, including breeders and offspring, had a radio frequency identification (RFID) tag implanted under its skin for identification purposes. Adult rats (PND 65-90) of both sexes only participated in oxycodone self-administration experiments. A separate group of adolescent rats (PND38-44) underwent all behavioral tests (i.e. open field, novel object interaction, elevated plus maze). Experiments were conducted on these two separate cohorts to avoid the potentially confounding effects that extensive behavioral testing might have prior to the initiation of the lengthy oxycodone SA protocol. The Animal Care and Use Committee of The University of Tennessee Health Science Center approved all procedures, which complied with NIH Guidelines for the Care and Use of Laboratory Animals. Animals were sacrificed by thoracotomy under deep isoflurane anesthesia.

### Drugs

Oxycodone HCl, a kind gift by Noramco (Wilmington, DE), was dissolved in distilled water.

### Open field test (OFT)

OFT was carried out as previously reported between postnatal days 30–32 [19]. Two OFT arenas were constructed using black acrylic glass, measuring 100 cm (L) ×  100 cm (W) ×  50 cm (H), which were placed side by side. The floors were covered by wood boards painted with either black or white acrylic paint (ART-Alternatives, ASTM D-4236, Emeryville, CA, USA) to contrast the coat of the animals (i.e., a black board was used for rats with white fur). The test chambers were illuminated by a long-range, 850-nm infrared light (LIR850-70, LDP LLC, Carlstadt, NJ) located 160 cm above the center of the two test chambers. No source of visible light was present during behavioral testing, with the exception of a flat panel monitor (Dell 1908FP). A digital camera (Panasonic WV-BP334) fitted with an 830 nm infrared filter (X-Nite830-M37, LTP LLC, Carlstadt, NJ) and located next to the infrared light source was used to record the behavior of the rats. All rats were released at the same corner of the test chamber, and data were collected for 20 min.

### Novel object interaction (NOI) test

This test was conducted the day after the OFT in the same arena. A cylindrical rat cage constructed using 24 aluminum rods (30 cm in length) spaced 1.7 cm apart was used as the novel object [20]. The bottom and top of the cage (15 cm in diameter) were manufactured using a 3D printer from polylactic acid. The novel object was placed in the center of the arena before testing. The test duration was 20 min and was recorded using the same camera as that used in the OFT.

### Elevated plus maze (EPM)

EPM was tested the day after NOI. The maze was constructed using black acrylic glass. The floors of the maze were covered by wood boards painted with black or white acrylic paint. The platform was 60 cm above the floor, with all four arms measuring 12 cm (W) ×  50 cm (L). The two opposing closed arms had walls measuring 30 cm (H). Rats were placed into the center of the maze facing the closed arm. The behavior of the rat was recorded for 6 min using the digital video system described above.

### Analysis of video data

Ethovision XT video tracking system (RRID:SCR_000441, Version 15.0, Noldus Information Technology, The Netherlands) was used to analyze the videos recorded in all behavioral tests. After identifying the arena and calibrating the size of the arena, specific zones in the arena were outlined. For OFT and NOI, one center zone, which was a circular region with a diameter of 20 cm, was used. The extracted data included the total distance traveled in the arena, the duration and the frequency the test rat was present in specific zones, and the distance of the subject to the zones. The center of the subject rat was used for all calculations.

### Oral operant oxycodone self-administration

We employed the same methodology as previously reported [8], with minor adjustments. The operant chamber (Med Associates) featured two lickometers: licks on the active spout following a fixed ratio 5 (FR5) schedule triggered the immediate release of a 60 μl oxycodone solution (0.025–0.10 mg/ml) onto the spout’s tip, along with the activation of an LED visual cue. A 20-second timeout followed the drug delivery, during which licks on the active spout and any licks on the inactive spout were recorded but had no programmed consequences. Rats had free access to food and water and were kept under a reversed light cycle, being tested in their dark phase.

**Fig 1:**
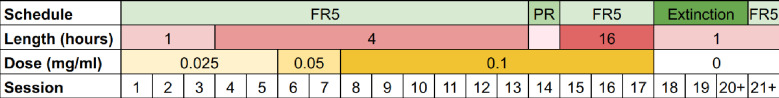
Protocol and schedule of operant oxycodone self-administration. The first three sessions were conducted daily. Subsequent sessions were conducted on alternate days. The final scheduled FR5 session was the reinstatement of extinguished oxycodone seeking.

Training commenced with three daily 1-h FR5 sessions at 0.025 mg/ml oxycodone concentration. Subsequent sessions extended to 4-h and occurred every other day. From session four onwards, we doubled the dose every two sessions up to the maximum dose of 0.1 mg/ml. Rats underwent six sessions at this highest dose, followed by a progressive ratio test on session fourteen. During the progressive ratio (PR) test, the number of licks to obtain a subsequent reward was determined using the exponential formula 5e^0.2 × injections −  5^, such that the required responses per injection were as follows: 1, 2, 4, 9, 12, 15, 20, 25, 32, 40, 50, etc [21]. The PR sessions ended after 20 min of inactivity. The final ratio completed is the breakpoint. Session lengths then increased to 16-h (4 PM - 8 AM) for three sessions. . Extinction sessions were conducted for 1-h daily, without programmed consequences, until licks on the active spout decreased to less than fifty for two consecutive sessions, when, in general, the number of licks on the active vs inactive sprouts were no longer significantly different. A final reinstatement session was carried out where active spout licks triggered only visual cue delivery without oxycodone. Final reinstatement data were not collected on a few strains due to campus closures from unexpected weather conditions. We recorded both the number and timing of oxycodone deliveries as well as licks on active and inactive spouts. The procedure is summarized in Fig 1. Full strain names and sexes of rats involved in oxycodone self-administration, along with the number of rats per strain (minimum is 3) and sex are listed in Table 1.

**Table 1:** The number of rats per strain for each sex.

Strain	Female	Male
ACI/EurMcwi	9	6
BDIX/NemOda	4	
BN-Lx/CubMcwi	5	3
BN/NHsd	6	
BN/NHsdMcwi	4	3
BUF/Mna	3	
BXH2/CubMcwi	7	7
BXH6/CubMcwi	10	5
F344/DuCrl	6	4
F344/NHsd	6	
F344/StmMcwi	8	7
FHH/EurMcwi	5	4
FXLE15/StmMcwi	7	8
FXLE19/StmMcwi	5	
HXB10/IpcvMcwi	10	10
HXB10XWMI	7	7
HXB2/IpcvMcwi	0	7
HXB23/IpcvMcwi	5	6
HXB31/IpcvMcwi	11	9
HXB4/IpcvMcwi		5
LE/StmMcwi	5	4
Lew/NHsd	8	8
LEXF2B/StmMcwi	3	
LEXF5/StmMcwi		5
M520/NMcwi	6	3
MWF/Mcwi	5	4
SHR/NCrl	6	
SHR/OlaIpcvMcwi	6	4
SHR/OlalpcvxBN/NHsdMcwi	4	
SR/JrHsd	3	
SS/JrHsdMcwi	8	12
SS/JrHsdMcwiCrl	6	
SS/JrHsdMcwixSHR/Olalpcv	9	7
WAG/RijCrl	4	7
WLI/Eer	6	3
WMI/Eer	6	5

### Identification of augmenter vs non-augmenter strains

In each sex, HRDP strains with more than a threefold increase in oxycodone (0.1 mg/ml) intake during 16-h vs 4-h sessions were classified as augmenters: 7 females, FXLE19/Stm, WAG/RijCrl, SR/JrHsd, LEXF2B/Stm, (SHR/OlaIpcv x BN/NHsdMcwi)F1, FXLE15/Stm, BDIX/NemOda; 6 males, F344/Stm, LEXF5/Stm, FXLE15/Stm, BXH2, LE/Stm, M520/NMcwi). In each sex, all HRDP strains were then ranked by intake in 4-h sessions. For each augmenter strain, one strain that ranked immediately above it (i.e., higher 4-h intake) and not already identified as an augmenter or non-augmenter was specified as the control non-augmenter strain. These are: females, SHR/OlaIpcv, BXH2, BN/NHsdMcwi, HXB23, HXB31, F344/Stm, M520/NMcw; males, (SS/JrHsdMcwi x SHR/Olalpcv) F1, BXH6/Cub, F344/DuCrl, BN-Lx/Cub, HXB31, HXB23.

### Allele frequencies of oxycodone metabolism enzymes in augmenter vs non-augmenter strains

Genetic variants (single nucleotide polymorphisms, SNPs) for the HRDP were sourced from a recent study [14]. We identified 1,263 SNPs in the Cyp3a2, Cyp3a9, and Cyp2d1 genes (including exons, introns, and adjacent intergenic regions). In each sex, Fisher’s exact test was applied to compare the allele frequencies of all SNPs between Augmenters and Non-augmenters. The raw p-values were then adjusted using the false discovery rate.

### Estimate of heritability

The between-strain variance provides a measure of additive genetic variation (VA), while within-strain variance represents environment variability (VE). An estimate of narrow-sense heritability (i.e. the proportion of total phenotypic variation that is due to the additive effects of genes, *h*^2^) for oxycodone reward was obtained using the formula: h2 =½* VA/( ½ * VA+VE) [22,23].

### Statistical analysis of behavioral data

The number of licks on active and inactive spouts was transformed into log scale to fit a normal distribution. The number of licks, reward, and intake during acquisition were analyzed by repeated measures ANOVA, where session and spouts were treated as within subject variables. Post-hoc tests were conducted using the Tukey HSD procedure. Phenotypic correlations were determined using the Pearson test. Data were expressed as mean ±  SEM. Statistical significance was assigned at p <  0.05. All analyses were conducted using R statistical language. Data and matched analytic tools are available in FAIR format at www.GeneNetwork.org (see *Data Availability*).

## Results

### Operant oral oxycodone self-administration in males and females

Our operant conditioning protocol reinforces the licking of a drug delivery spout by delivering one drop (60 μl) of oxycodone under a fixed ratio 5 (FR5, with a 20s time out period) schedule. The ~ 65 day procedure is summarized in Table 1 of Methods.

**Fig 2:**
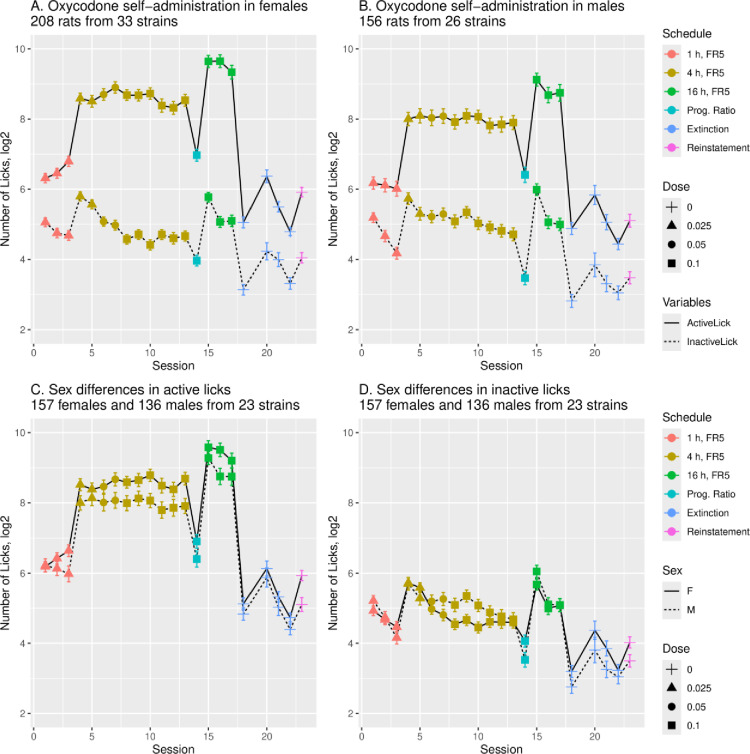
Number of licks during oxycodone SA. Rats showed a strong preference for the active over inactive spout in females (A) and males (B) throughout the HRDP. (C) The number of licks on the active spout was greater in females (n =  157) than in males (n = 136) during both 4-h (p =  0.003) and 16-h sessions (p =  0.03) in the 23 strains where both sexes were studied. (D) The number of licks on the inactive spout was not different between sexes (p > 0.05) during either the 4-h or 16-h sessions.

Of 36 strains included in the hybrid rat diversity panel (HRDP; [13]), oral operant oxycodone SA was studied in females and males from 33 and 26 strains, respectively (Fig 2A and 2B). The number of active (mean±sem) and inactive licks emitted by rats across all strains are shown for operant sessions that increased in duration (1-h/4 to–h/16-h) and dose (0.025–0.10 mg/kg) followed by four extinction sessions on alternating days and reinstatement of extinguished drug seeking behavior. A progressive ratio schedule of oxycodone 0.1 mg/kg was conducted on the day prior to the first 16-h session. In both sexes (Fig 2), a strong preference for the active vs. inactive spout was observed across the HRDP at all doses and session durations. In the 23 strains common to both sexes, active licks were greater in females than males during both 4-h and 16-h sessions (panel C: 4-h, F_1,275_ =  9.14, p =  0.003; 16-h, F_1,233_ =  4.97, p =  0.03). Inactive licks were not different between sexes (panel D: 4-h, F_1,275_ = 2.7, p > 0.05; 16-h, F_1,233_ =  0.76, p > 0.05).

**Fig 3:**
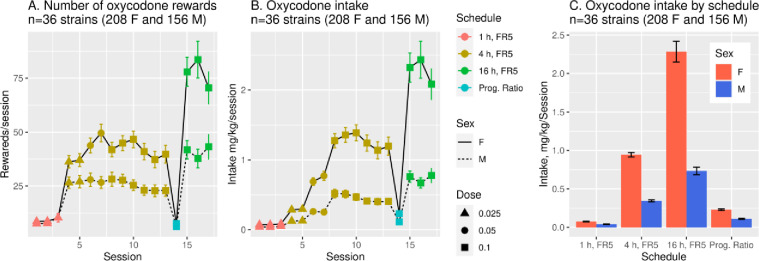
Sex differences in oxycodone rewards and intake. The mean oxycodone reward (A) and intake (B) per session (FR5) were significantly greater in females than males in the 23 strains where both sexes were studied (p < 0.001 for both). Intake was also significantly greater in females than in males across different stages (C) of SA (p < 0.001 for all). In both sexes, the greatest intake occurred during 16-h sessions.

There was a strong sex difference in oxycodone intake across the 23 strains common to both sexes (Fig 3). In females, across all SA sessions, mean rewards/session (panel A: F_1,275_ =  125.9, p < 2e-16) and intake/session (B: F_1,275_ =  105.2, p < 2e-16) were greater than in males. Similarly, there were sex differences in oxycodone intake across different SA stages (panel C), including 1-h sessions (F_1,275_ = 66.8, p =  1.1e-14), 4-h sessions (F_1,275_ =  119.9, p < 2e-16), and 16-h sessions (F_1,233_ =  79.7, p < 2e16). In both sexes given access to the same dose of oxycodone (0.1 mg/ml) during 4-h and 16-h sessions, the greatest intake occurred during the long 16-h sessions: intake increased in both males (F_1,753_ = 83.6, p =  2e-16) and females (F_1,901_ =  162.9, p =  2e-16).

**Fig 4:**
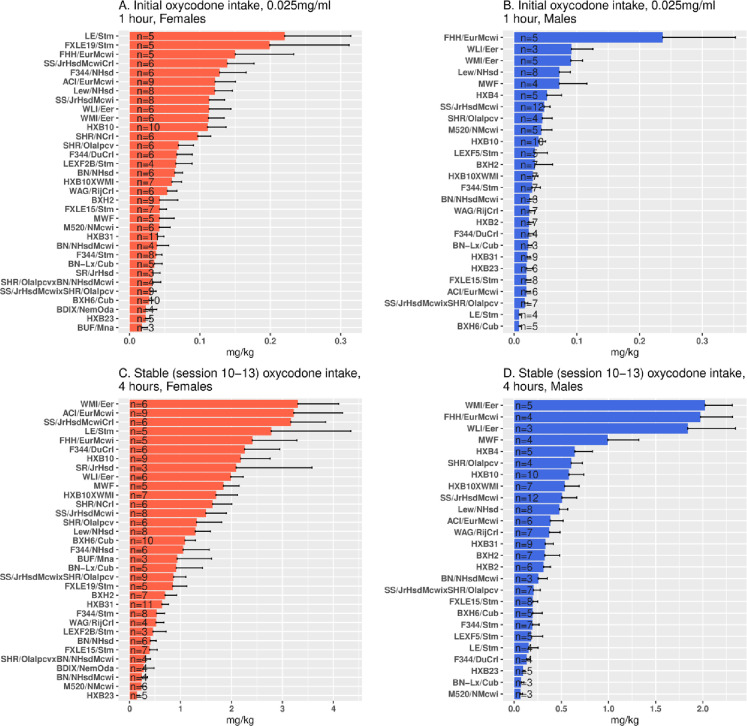
Initial 1-h and stable 4-h oxycodone intake within each sex across the 36 HRDP strains. Mean intake during the first three sessions (top 2 panels) showed large strain differences in each sex. Similarly, mean intake during the last three 4-h sessions (stable intake; lower 2 panels) varied across the HRDP in each sex. (note: the difference in X axis scales).

Across the 36 HRDP strains, oxycodone intake was strain-dependent in both sexes (Fig 4). Mean intake during the first three 1-h sessions, when SA of oxycodone (0.025 mg/ml) was acquired (panels A. B), varied greatly by strain (male: F_25,432_ =  9.07, p =  9.5e-27. female: F_32,570_ = 7.45, p =  5.3e-27). Similarly, mean intake during the final three 4-h sessions, when stable oxycodone (0.1 mg/ml) intake was attained (panels C, D), varied greatly by strain in both sexes (F_25,506_ =  25.74, p =  3.8e-74 in males and F_32,620_ =  9.59, p =  1.30e-36 in females).

### Stable Oxycodone Intake during 4-h Limited Access vs.16-h Extended Access Sessions

**Fig 5:**
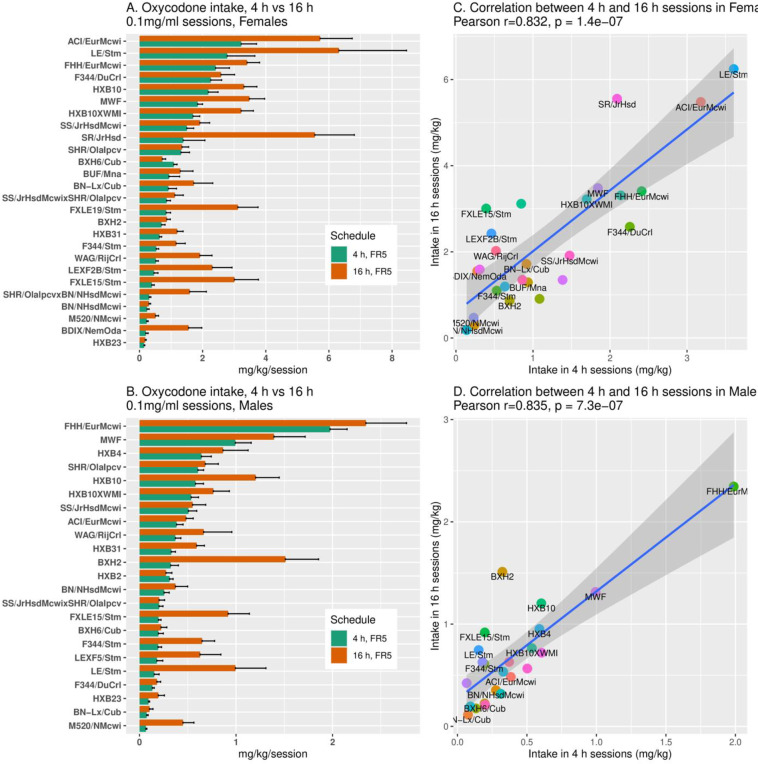
Oxycodone (0.1 mg/kg) intake in 4-h and 16-h sessions within each sex. A, B: Extending access from 4-h to 16-h increased total drug intake/session in many strains and both sexes. Within specific strains, increased 16-h intake was sex-specific. C, D: The correlation of oxycodone intake between 4 h and 16 h sessions was significant in both females (r =  0.832, p < 0.0001) and males (r =  0.835, p < 0.0001).

We compared and correlated stable oxycodone intake (0.1 mg/ml) during limited 4-h vs. extended 16-h sessions within each sex across the 36 HRDP strains. Oxycodone intake in 16-h sessions exceeded 4-h sessions in many strains (Fig 5A and 5B). This change was sex-specific in that specific strains showed increased 16-h consumption in only one sex of the 23 common strains. In addition, a greater number of strains throughout HRDP showed increased 16-h oxycodone intake in males compared to females. Fig 5C and 5D show the correlations within females (r =  0.832, p < 0.0001) and within males (r =  0.835, p < 0.0001) respectively, for oxycodone intake in 4-h vs. 16-h sessions across all strains. In summary, in both sexes across the HRDP, oxycodone intake during 4-h sessions was predictive of intake during 16-h sessions. Within sex, the fold-increase in oxycodone intake during our 65-day SA protocol, from the initial 1-h sessions at 0.025 mg/ml to the final 16-h sessions at 0.1 mg/ml, varied greatly by strain. In males, the increase ranged from 4.67 (BN-Lx/CubMcwi) to 126.0-fold (LE/StmMcwi); in females, it was 7.2 (BN/NHsdMcwi) to 167.2-fold (SR/JrHsdMcwi).

### Augmented Oxycodone Intake During Long Access Sessions in a Subset of Strains

**Fig 6:**
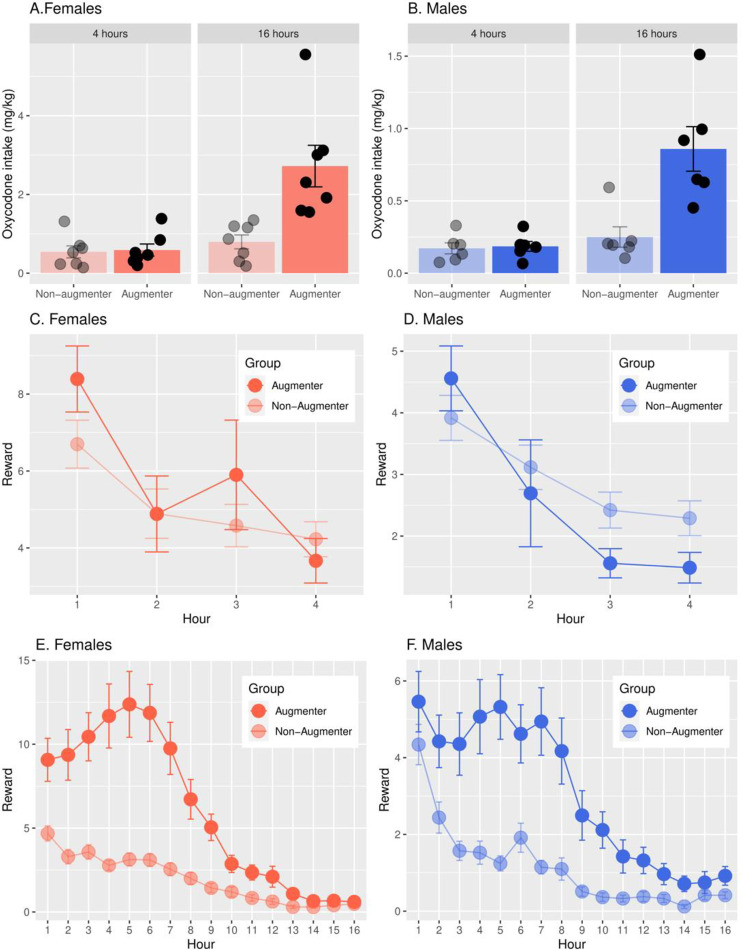
Augmented oxycodone intake in 16-h vs 4-h sessions. A subset of strains (7 female, 6 male) augmented their oxycodone intake during long access 16-h vs 4-h sessions by at least 3-fold (Females: F_1,12_ = 15.03, p = 0.002; Males: F_1,10_ =  18.22, p =  0.002, panels B). Inter-sex comparisons were not conducted because only three strains were common to both sexes. At 4-h, non-augmenters and augmenters had similar oxycodone intake within each sex (Females; F_1,12_ =  0.05, p =  0.84, panels A; Males: F_1,10_ =  0.08, p =  0.79, Panel B). In the 4-h sessions (panels C, D), maximum oxycodone intake occurred in both sexes of augmenters and non-augmenters during the first hour and declined thereafter toward baseline levels by 3-4 hours. In the 16-h sessions (panels E, F), intake in non-augmenters was maximal at 1 hour, declining gradually thereafter in both sexes. In contrast, early intake, from 1-6 hours, was much greater in the augmenters of both sexes, rapidly decreasing from approximately 7-11 hours, and gradually thereafter. Augmenter types: 7 females, FXLE19/Stm, WAG/RijCrl, SR/JrHsd, LEXF2B/Stm, (SHR/OlaIpcv x BN/NHsdMcwi) F1, FXLE15/Stm, BDIX/NemOda; 6 males, F344/Stm, LEXF5/Stm, FXLE15/Stm, BXH2, LE/Stm, M520/NMcwi). Control non-augmenter types: females, SHR/OlaIpcv, BXH2, BN/NHsdMcwi, HXB23, HXB31, F344/Stm, M520/NMcw; males, (SS/JrHsdMcwi x SHR/Olalpcv) F1, BXH6/Cub, F344/DuCrl, BN-Lx/Cub, HXB31, HXB23.

Across all strains, each sex increased intake during 16-h vs 4-h sessions (p < 2e-16), but a subset of the 23 strains dramatically augmented (>3-fold) their intake at 16-h (Fig 6A and 6B; F_1,12_ =  15.03, p =  0.002 for females and F_1,10_ =  18.22, p = 0.002 for males). In contrast, oxycodone intake at 4-h was similar in augmenters vs non-augmenters within each sex (Fig 6A and 6B; female, F_1,12_ =  0.05, p =  0.84; male, F_1,10_ =  0.08, p =  0.79). Inter-sex comparisons were not conducted because only 3 strains were common to both sexes. Fig 6C–6F is a high time resolution view of oxycodone intake by hour and sex during 4-h and 16-h sessions. In 4-h sessions (Fig 6C and 6D), maximum oxycodone intake occurred in both sexes of augmenters and non-augmenters during the first hour and declined thereafter toward baseline levels by 3-4-h. In 16-h sessions (Fig 6E and 6F), intake in non-augmenters was maximal at 1-h, declining gradually thereafter in both sexes. In contrast, early intake, from 1-6-h, was much greater in the augmenters of both sexes, rapidly decreasing from approximately 7-11-h, and gradually thereafter. By 13-16-h, intake within sex was similar in both augmenters and non-augmenters. Augmenters of both sexes have a prolonged interval of loading-up with oxycodone from approximately 1-6-h that is not observed in non-augmenters. Following this interval, oxycodone intake declines steadily, devoid of periodic increases.

To examine whether genetic differences in oxycodone metabolism might contribute to the divergent patterns of 16-h oxycodone intake in augmenters vs non-augmenters, we identified 1,263 SNPs in different alleles associated with the three major metabolic enzymes (i.e., CYP3A2, CYP3A9, and CYP2D1) that differed between these two groups. The allele frequencies of these SNPs were not different statistically, even at 30% false discovery rate. While these data suggest that genetic variation in isoenzymes modulating oxycodone metabolism is unlikely responsible for the divergent patterns of 16-h oxycodone intake in augmenters vs non-augmenters, they do not eliminate the possibility of variation in gene expression by other mechanisms (e.g., transcriptional or post-translational). Additionally, it is important to note that this analysis did not examine haplotypes, which could capture combined effects of multiple variants and provide further insights into regulatory mechanisms.

### Progressive ratio schedule and reinstatement of extinguished oxycodone seeking

**Fig 7:**
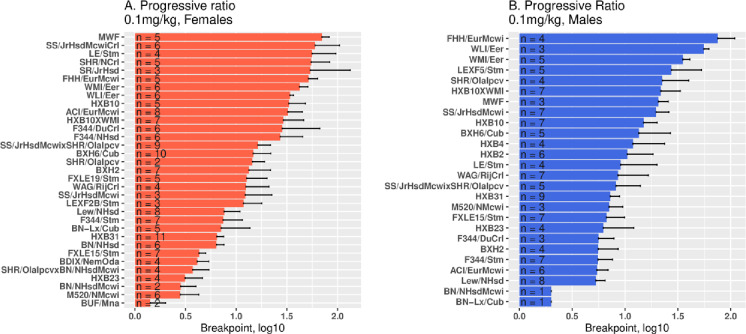
Breakpoints during progressive ratio schedule of increasing active licks per oxycodone dose (0.1 mg/kg). In each sex, the breakpoints reached were significantly strain-dependent (A. females, F_32,148_ =  5.2, **p** =  1.8e-12; B. males, F_25,103_ =  2.6, **p** =  0.0003) and greater in females than males (F_22,227_ =  6.4, **p** =  2.5e-14). X-axis is in log scale.

In Fig 7, the breakpoints achieved were strain-dependent in each sex (A. females, F_32,148_ =  5.2, p =  1.8e-12; B. males, F_25,103_ = 2.6, p =  0.0003). In the subset of 23 strains where PR was measured in both sexes, the effect of sex was also significant (F_22,227_ =  6.4, p =  2.5e-14). Therefore, in both sexes, the motivation to obtain oral oxycodone is strain- and sex-dependent.

**Fig 8:**
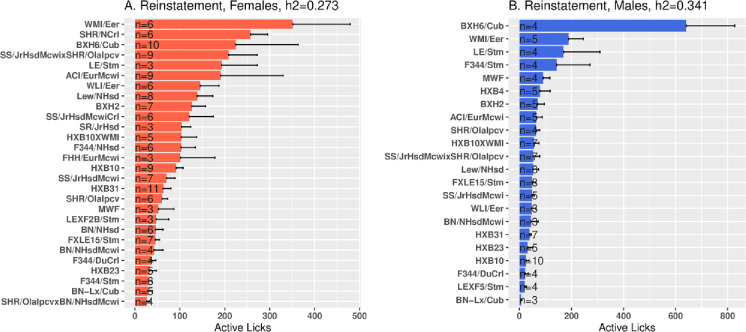
Cue-induced reinstatement of extinguished oxycodone seeking. Reinstatement was conducted after the number of licks on the active spout was less than 100 during two consecutive extinction sessions. There was a strain difference in the number of active licks during reinstatement in females (A. F_31,142_ =  2.9, p =  1.3e-5) and males (B. F_25,94_ =  3.2, p =  2.0e-5). In the 23 strains where reinstatement was studied in both sexes, female active licks were greater (F_23,221_ =  3.8, p =  1.5e-7).

During cue-induced reinstatement of extinguished oxycodone seeking (Fig 8), we found a strain difference throughout the HRDP in the number of active licks in females (A. F_31,142_ =  2.9, p =  1.3e-5) and males (B. F_25,94_ =  3.2, p =  2.0e-5). In the 23 strains where reinstatement was studied in both sexes, female active licks were greater (F_23,221_ =  3.8, p =  1.5e-7). Hence, cue-induced oxycodone-seeking is strain-dependent and stronger in females than males.

### The correlation between Male (M) and Female (F) oxycodone intake

**Fig 9:**
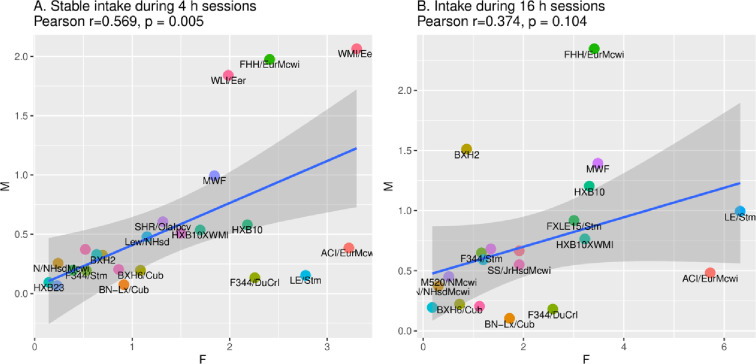
In the 23 common strains, the correlation in male (M) vs. female (F) oxycodone (0.1 mg/kg) intake/session during 4-h and 16-h sessions. A. During 4-h sessions with stable oxycodone intake, M vs F intake was highly correlated across the HRDP (Pearson r =  0.54, p =  0.008). B. However, during 16-h sessions, intake between sexes was not significantly correlated (r =  0.35, p =  0.104).

During 4-h sessions with stable oxycodone intake, male vs. female intake was highly correlated across strains (Fig 9A, Pearson r =  0.57, p =  0.005). However, during 16-h sessions, intake was weakly correlated across the HRDP (Fig 9B, r =  0.37, p =  0.104). The heritability of oxycodone intake declined in males from 4-h to 16-h sessions (Table 2; *h*^2^ 0.63 to 0.22) and this also occurred, to some extent, in females (*h*^2^ 0.38 to 0.30); this decrease indicated increased intra-strain variability in intake during the extended access sessions, which could reduce the correlation between sexes (Fig 8B).

**Table 2:** In the 23 common strains, the correlation between males and females in oxycodone self-administration.

Stage	Phenotype	Pearson r	p	Significance
FR5, 0.025mg, 1h	ActiveLick	0.185	0.3986	
	InactiveLick	0.563	0.0051	^**^
	Reward	0.514	0.0120	^*^
	Intake	0.402	0.0573	
FR5, 0.025mg, 4h	ActiveLick	0.298	0.1666	
	InactiveLick	0.331	0.1227	
	Reward	0.674	0.0004	^***^
	Intake	0.589	0.0031	^**^
FR5, 0.05mg, 4h	ActiveLick	0.419	0.0464	^*^
	InactiveLick	0.188	0.3901	
	Reward	0.713	0.0001	^***^
	Intake	0.629	0.0013	^**^
FR5, 0.1mg, 4h	ActiveLick	0.435	0.0380	^*^
	InactiveLick	0.254	0.2422	
	Reward	0.768	0.0000	^***^
	Intake	0.690	0.0003	^***^
FR5, 0.1mg, 4h Stable	ActiveLick	0.439	0.0362	^*^
	InactiveLick	-0.090	0.6831	
	Reward	0.653	0.0007	^***^
	Intake	0.567	0.0047	^**^
FR5, 0.1mg, 16h	ActiveLick	0.328	0.1578	
	InactiveLick	0.361	0.1182	
	Reward	0.441	0.0513	
	Intake	0.374	0.1044	
Progressive Ratio	ActiveLick	0.426	0.0429	^*^
	InactiveLick	0.169	0.4405	
	BreakPoint	0.649	0.0008	^***^
	Intake	0.493	0.0168	^*^
Extinction Day1	ActiveLick	0.239	0.3112	
	InactiveLick	0.125	0.6009	
Extinction Last Day	ActiveLick	0.300	0.1858	
	InactiveLick	0.045	0.8478	
Reinstatement	ActiveLick	0.517	0.0138	^*^
	InactiveLick	0.217	0.3313	
Sum of	ActiveLick	0.302	0.1613	
All Sessions	InactiveLick	0.314	0.1439	
	Reward	0.587	0.0032	^**^
	Intake	0.402	0.0574	

In Fig 9A, 4-h intake was highly correlated between sexes across the 23 strains, despite strong sex differences in oxycodone intake (Fig 3), motivation to obtain drug as measured by the breakpoint in progressive ratio tests (Fig 7), and seeking behavior during reinstatement (Fig 8). This demonstrates common genetic modulation of variation in both sexes. Table 2 underscores this strong strain-dependent genetic control: multiple parameters of oxycodone self-administration were well correlated between sex across the 23 strains. These include: oxycodone intake during 1-h, 4-h and 16-h sessions, effort to obtain reward (i.e., breakpoint and rewards obtained during PR trial), and active licks during reinstatement.

### The Heritability (*h* ^2^) of oxycodone behavioral phenotypes

Table 3 shows the heritability (*h*^2^) of oxycodone SA parameters by sex. Active licks, reward, and intake show h^2^ values associated with significant differences between strains for all SA parameters in each sex. Inactive licks were generally at much lower h^2^ values in each sex. In males, the *h*^2^ for 4-h oxycodone intake (mg/kg b.wt./session) was consistently greater than in females by approximately 50%. In contrast, *h*^2^ for active licks in 4-h sessions was higher in females. In 16-h sessions, the *h*^2^ for both intake and active licks was greater in females than males. Additionally, the female *h*^2^ for intake under a. progressive ratio schedule was 0.41 vs. 0.31 in males; active licks under this schedule showed a greater divergence in *h*^2^ between female and male: 0.46 and 0.15, respectively.

**Table 3:** Heritability and ANOVA results for oxycodone SA parameters.

Stage	Phenotype	Males				Females			
		h2	Df	F	p	h2	Df	F	p
FR5, 0.025mg, 1h	Active Lick	0.30	(25,441)	7.77	1.3E-22	0.37	(32,588)	10.87	2.2E-41
	Inactive Lick	0.22	(25,436)	5.20	8.4E-14	0.23	(32,579)	5.89	3.0E-20
	Reward	0.34	(25,441)	9.13	4.9E-27	0.26	(32,588)	6.99	4.0E-25
	Intake	0.35	(25,432)	9.07	9.5E-27	0.29	(32,570)	7.45	5.3E-27
FR5, 0.025mg, 4h	Active Lick	0.30	(25,277)	5.34	3.3E-13	0.41	(32,376)	8.71	2.9E-29
	Inactive Lick	0.08	(25,272)	1.86	9.0E-03	0.25	(32,366)	4.63	1.2E-13
	Reward	0.44	(25,277)	9.15	3.8E-24	0.34	(32,376)	6.75	5.2E-22
	Intake	0.44	(25,275)	8.95	1.4E-23	0.30	(32,374)	5.65	9.8E-18
FR5, 0.05mg, 4h	Active Lick	0.30	(25,276)	5.31	4.2E-13	0.47	(32,374)	10.82	1.9E-36
	Inactive Lick	0.16	(25,276)	2.84	1.6E-05	0.23	(32,358)	4.11	1.5E-11
	Reward	0.66	(25,278)	20.38	5.2E-49	0.42	(32,374)	8.95	4.5E-30
	Intake	0.65	(25,278)	20.12	1.6E-48	0.41	(32,374)	8.59	9.0E-29
FR5, 0.1mg, 4h	Active Lick	0.30	(25,277)	5.23	7.0E-13	0.53	(32,372)	13.40	1.7E-44
	Inactive Lick	0.07	(25,275)	1.78	1.4E-02	0.16	(32,363)	2.96	4.5E-07
	Reward	0.66	(25,277)	20.93	6.3E-50	0.38	(32,372)	7.65	2.5E-25
	Intake	0.65	(25,277)	20.17	1.5E-48	0.37	(32,372)	7.36	2.8E-24
FR5, 0.1mg, 4h Stable	Active Lick	0.39	(25,506)	10.17	2.9E-31	0.51	(32,620)	15.33	4.4E-59
	Inactive Lick	0.21	(25,505)	4.65	4.2E-12	0.25	(32,615)	5.53	7.9E-19
	Reward	0.64	(25,506)	26.86	1.1E-76	0.37	(32,620)	9.10	1.5E-34
	Intake	0.63	(25,506)	25.74	3.8E-74	0.38	(32,620)	9.59	1.3E-36
FR5, 0.1mg, 16h	Active Lick	0.25	(22,352)	5.60	2.0E-13	0.40	(25,426)	10.92	2.1E-32
	Inactive Lick	0.15	(22,350)	3.41	7.0E-07	0.12	(25,425)	3.10	1.4E-06
	Reward	0.25	(22,352)	5.65	1.4E-13	0.29	(25,426)	7.25	1.0E-20
	Intake	0.22	(22,352)	4.94	1.9E-11	0.30	(25,426)	7.44	2.3E-21
Progressive Ratio	Active Lick	0.15	(23,103)	1.82	2.2E-02	0.46	(29,145)	5.42	2.7E-12
	Inactive Lick	0.08	(23,101)	1.41	1.2E-01	0.21	(29,145)	2.41	3.4E-04
	BreakPoint	0.26	(23,103)	2.64	4.6E-04	0.44	(29,145)	5.10	1.7E-11
	Intake	0.31	(23,103)	3.14	3.8E-05	0.41	(29,145)	4.60	3.6E-10
Extinction Day1	Active Lick	0.32	(21,99)	3.24	4.4E-05	0.30	(25,125)	3.20	1.0E-05
	Inactive Lick	0.14	(21,99)	1.80	2.9E-02	0.11	(25,125)	1.61	4.6E-02
Extinction Last Day	Active Lick	0.22	(20,81)	2.26	5.4E-03	0.28	(25,127)	2.95	3.9E-05
	Inactive Lick	0.04	(20,81)	1.17	3.1E-01	0.22	(25,127)	2.46	5.9E-04
Reinstatement	Active Lick	0.34	(21,92)	3.41	2.5E-05	0.27	(27,140)	2.94	2.0E-05
	Inactive Lick	0.07	(21,92)	1.35	1.7E-01	0.26	(27,140)	2.76	5.9E-05
Total	Active Lick	0.22	(25,2395)	22.84	7.3E-93	0.31	(32,3097)	37.86	3.0E-195
	Inactive Lick	0.12	(25,2380)	11.63	4.5E-44	0.14	(32,3047)	14.40	2.0E-71
	Reward	0.33	(25,2397)	39.14	8.6E-158	0.18	(32,3097)	19.50	5.1E-100
	Intake	0.20	(25,2386)	20.14	2.4E-81	0.15	(32,3077)	15.21	4.8E-76

Overall, the relative degree of oxycodone heritability in males vs. females was specific for each behavioral phenotype. Oxycodone intake in 4-h sessions at three doses showed the highest heritability and some of the largest differences between sexes, with males considerably greater than females. However, active licks in 4-h sessions and both intake and active licks in 16-h sessions and under a PR schedule were greater in females than males.

### Correlations between behavioral tests and oxycodone SA

We assessed anxiety-like response to novelty using elevated plus maze (EPM), open field test (OFT), and novel object interaction (NOI) in *naive* rats (strain means by sex are provided in Supplementary Table 1). Table 4 shows that the heritability (h^2^) of behavioral test parameters (i.e., 5 EPM traits, 4 OFT, 4 NOI) varied within each sex across the HRDP strains we tested (n =  19 female, 17 male). Within sex, the most heritable trait was total distance in all three behavioral tests (range across tests by sex: female, 0.51–0.58; male, 0.24–0.49).

**Table 4:** Heritability and ANOVA for comparison across HRDP of anxiety-like and novelty response traits in naive inbred rats.

Behavior Test	Trait	Males				Females			
		h2	Df	F	p	h2	Df	F	p
OFT	Total Distance	0.49	(15,96)	6.41	3.19E-09	0.59	(15,105)	10.06	2.19E-14
OFT	Distance to arena center	0.55	(15,96)	7.87	3.26E-11	0.44	(15,105)	5.98	7.96E-09
OFT	Duration in arena center	0.33	(15,96)	3.69	4.44E-05	0.12	(15,105)	1.90	3.07E-02
OFT	Center frequency	0.33	(15,96)	3.79	3.09E-05	0.40	(15,105)	5.19	1.32E-07
NOI	Total Distance	0.40	(15,100)	4.89	4.74E-07	0.56	(16,114)	9.06	5.71E-14
NOI	Distance to arena center	0.28	(15,100)	3.25	2.13E-04	0.25	(16,114)	3.07	2.54E-04
NOI	Duration in arena center	0.11	(15,100)	1.69	6.40E-02	0.15	(16,114)	2.13	1.15E-02
NOI	Center frequency	0.27	(15,100)	3.20	2.58E-04	0.49	(16,114)	7.03	5.35E-11
EPM	Total distance	0.24	(16,88)	2.75	1.31E-03	0.51	(18,101)	6.69	1.12E-10
EPM	Duration in open arm	0.19	(16,88)	2.34	6.27E-03	0.22	(18,101)	2.50	2.04E-03
EPM	Frequency in open arm	0.30	(16,88)	3.37	1.29E-04	0.24	(18,101)	2.74	7.68E-04
EPM	Duration in closed arm	0.30	(16,88)	3.42	1.08E-04	0.12	(18,101)	1.73	4.60E-02
EPM	Frequency in closed arm	0.23	(16,88)	2.64	2.01E-03	0.34	(18,101)	3.80	8.58E-06

**Fig 10:**
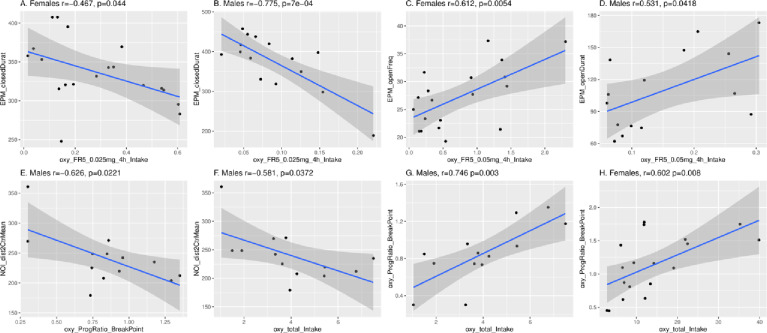
Representative correlations between oxycodone SA parameters and behavioral traits. Panels A-D show significant correlations for EPM vs oxycodone intake in 4-h SA sessions at 0.25 and 0.5 mg/ml in female (panels A, C) and male (B, D) HRDP strains. Significant correlations in male HRDP strains between NOI (distance to center) and PR (breakpoint) or total oxycodone intake are in panels E and F, respectively. Correlations between NOI and PR or total intake were not significant in females (see supplementary S1 Fig). Panels G and H, show significant correlations between two SA parameters, PR and total intake, in males and females, respectively. P values were not adjusted for multiple comparisons.

**Fig 11:**
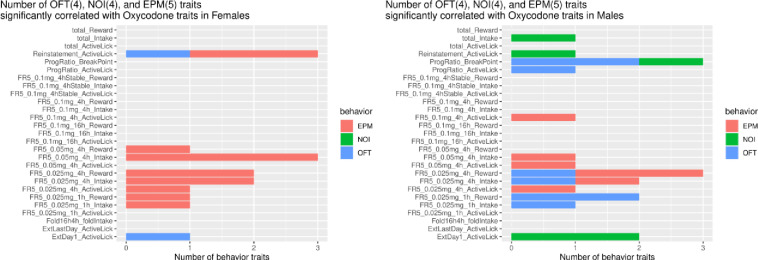
Behavioral traits that significantly correlate with oxycodone SA parameters. This bar graph groups all the behavioral traits measured in EPM, OFT, and NOI trials that were associated with each SA parameter; this grouping is based on the significant correlations (p  <  0.05) identified between a single behavioral trait and an SA parameter (supplementary data, S1 and S2 Figs).

We correlated multiple traits measured in each behavioral paradigm with 28 parameters of oxycodone SA, using the complete HRDP dataset. We used this dataset to find the maximum number of significant correlations within each sex with the proviso that deductions should not be drawn from inter-sex comparisons of the number of significant correlations. Fig 10 shows examples of two significant correlations for EPM within sex vs. 4-h intake of oxycodone 0.025 (panels A, B) and 0.05 (panels E, F). Fig 11 shows that EPM-defining traits (i.e., female dataset, 13; male dataset, 7) were associated with multiple SA parameters in each sex, and a large fraction of these associations were sex specific (e.g., female only 7/13). In females, three EPM traits (Fig 11) correlated with total 4-h oxycodone intake and one trait with 4-h intake in males. OFT traits were SA-associated in each sex: 8 in males and 2 in females, and specific SA associations were unique to each sex (Fig 11). NOI traits were associated with four SA parameters in males and none in females. Among these male NOI associations, NOI correlated with total oxycodone intake (i.e., sum of all doses at all time intervals in males/strain) and with active licks during reinstatement compared to OFT and EPM in reinstating females. Fig 10D shows the strain correlation for NOI (distance to center) and total oxycodone intake. At the highest dose of oxycodone (0.1 mg/kg), no behavioral traits correlated with intake, reward or active licks at 4-h and 16-h, barring one exception - active licks in males during 4-h sessions correlated with EPM. This is the same oxycodone dose consumed in the PR study that correlated with NOI and OF only in males.

### Correlation in Males between PR breakpoint, NOI (distance to center), and total oxycodone intake

NOI (distance to center) correlated with both PR breakpoint and total oxycodone intake in males, but not females (Fig 11); indeed, no behavioral traits were associated with either of these two SA parameters in females. Fig 10C shows this correlation for NOI and PR breakpoint in males. In Fig 10 (panels G, H), PR breakpoint also significantly correlated across strains with total oxycodone intake in both sexes. In summary, the following correlations were identified in males across strains: PR breakpoint x NOI (distance to center); total oxycodone intake x NOI; PR breakpoint x total oxycodone intake. Hence, these three independent measures are all significantly inter-correlated across strains only in males. This strongly suggests that CNS mechanisms governing novelty seeking and motivation to take oxycodone interact in males to regulate the total consumption of oxycodone.

## Discussion

There is substantial variation among humans in susceptibility to opioid addiction [24]. Outbred animal models also demonstrate substantial variation in responses to behavioral paradigms with face validity for important dimensions of human addiction [25]. We took advantage of the highly replicable behaviors among individuals within inbred rat strains to identify behavioral parameters of oxycodone SA that consistently varied across a large panel of inbred strains and several isogenic F1s—all members of the HRDP. All animals were bred, raised and tested in the same vivarium. Under these conditions, consistent strain variation in oxycodone SA will mainly reflect differences in the genetic control of SA variables. Indeed, we identified multiple heritable traits of oxycodone SA that also showed significant sex differences. The broad polygenic regulation of each behavioral parameter and the impact of subsets of variants on multiple traits implies a strong functional inter-dependency among these traits. Many of these SA variables correlated with the responses in naive HRDP rats to independent behavioral testing in EPM, NOI and OFT.

Oxycodone intake, both during initiation of SA and stable intake in 4-h sessions, was strain-dependent in both sexes (Fig 4) and heritable (Table 3). In the 23 strains common to both sexes, the mean amount consumed across all sessions (Fig 3) of increasing duration and dose was greater in females than males, and the mean number of active licks (Fig 2) was greater in females during 4-h and 16-h sessions. Similar to 4-h sessions, oxycodone intake in 16-h sessions was heritable in both sexes (Table 2). Within sex, oxycodone intake was correlated in 4-h vs. 16-h sessions (Fig 5C and 5D). Therefore, in individual female and male strains, intake in 4-h sessions predicted intake during extended access sessions. This within-strain correlation of oxycodone intake in 4-h and 16-h sessions is most probably controlled by the heritability of genes that regulate intake in both short and extended access sessions.

Oxycodone intake by a subset of strains in each sex was dramatically augmented (>3-fold) during 16-h sessions. Augmented intake was evident within the first 6-h and rapidly declined thereafter (Fig 6). Considering the half-life of blood oxycodone in rat [26], it is likely that an approximate doubling of blood levels occurred during this interval while intake and blood levels declined rapidly in non-augmenters. This suggests that oxycodone is either more rewarding in augmenters or that more drug is required to produce adequate reward, perhaps due to lower sensitivity or higher clearance. Since hourly and total intake in 4-h sessions were similar between augmenters and non-augmenters in both sexes (Fig 6), reduced sensitivity to the rewarding effects is unlikely in augmenters. Based on finding similar genetic variation in the three major enzymes controlling oxycodone metabolism, the enzymatic clearance of blood oxycodone is unlikely to vary between the augmenters and non-augmenters. However, the differential expression of the genes encoding these enzymes is a possibility, which might affect the metabolism of oxycodone. Overall, we suggest that oxycodone is probably more rewarding in augmenters due to the genetic control of reward efficacy.

In general, females across all strains manifest greater oxycodone intake in both short and extended access sessions and greater numbers of active licks. This strongly suggests the existence of a basic sex difference in the amount of oxycodone required to establish stable oral oxycodone reinforcement within the CNS. An extensive literature on sex differences in the sensitivity to opiate reinforcement [27,28] and analgesia supports this hypothesis.

Morphine has been reported to induce a more pronounced place preference in female Wistar rats at similar doses [27]. In self-administering female Sprague Dawley rats, more intravenous (i.v.) morphine and heroin were consumed and a broader range of doses were reinforcing than in males [28]. Moreover, at doses in the upper end of the dose-response range, morphine-induced place preference in females, but was no longer effective in males [29]. Both estrogen and progesterone receptors have been detected in dopamine terminals and medium spiny neurons within nucleus accumbens (NAc), which is involved in reward-associated learning and motivation to goal-oriented behaviors [30,31]. Estradiol rapidly enhanced NAc dopamine release and modulated dopamine binding, effects also observed in cycling females [30]. In mice, basal dopamine neuron activity and dopamine release in NAc were similar in both sexes, except during estrus when both increased [32]. Additionally, both systemic and intrastriatal estradiol rapidly amplified amphetamine-induced dopamine release in rat dorsal striatum [33]. In summary, rat models of opiate preference and operant self-administration demonstrate that opiates are more reinforcing in females, and at a broader and higher dose range. This is likely due, in part, to enhanced responsiveness to opiate-induced dopamine release under the influence of ovarian steroids. Hence, oral oxycodone appears to be more reinforcing in females across the HRDP, which drives greater licking behavior.

In humans, the patterns of opioid use and sensitivity to opioid dependency vary by sex. In comparison to men, women are more likely to be prescribed opioids and at higher doses, to use opioids for longer durations, and to develop dependence more rapidly [34,35]. The patterns of opioid use also differ by sex: prescription opioid misuse due to affective stress is more frequent in women compared to male misuse that is associated with behavioral and legal problems [36]. Women are also more likely to use prescription opioids upon first awakening in the morning [37].

PR breakpoints were higher in female strains across the HRDP. Similar differences have been reported for i.v. opiate SA in outbred rats [28]. Since PR breakpoint is a heritable SA parameter in both sexes, it is reasonable to expect significant inter-strain variability in the effect of sex if the overall effect of sex is small to moderate and the interaction between sex and heredity in each strain depends on the specific subsets of genes modulating PR in each strain.

Multiple traits measured during independent behavioral tests, conducted in naive rats, varied significantly across strains and were heritable (Table 4). These traits correlated with specific parameters of oxycodone SA, depending on sex (Fig 10 and supplementary S1, S2 Figs). EPM traits were associated with a common subset of SA parameters in both sexes, whereas NOI traits were associated with SA only in males. In both 4-h and 16-h sessions at high dose oxycodone, with one exception (Fig 10: male, EPM vs. active licks, 4-h), no SA parameters correlated with behavior. At high dose oxycodone, intake and active licks in short and long access sessions were not correlated with behavioral measures of anxiety (i.e., EPM and OFT) and novelty-seeking (i.e., NOI). Hence, the reinforcing efficacy of high dose oxycodone is unaffected by the intrinsic, strain-dependent level of anxiety or novelty-seeking associated with oxycodone intake at lower doses.

The NOI trait of distance to the center was inversely associated with both PR breakpoint and total oxycodone intake in males (Figs 9,10). In contrast, no correlations were found between these SA parameters and behavioral traits in females (Fig 11). Overall, the heritable, sex-specific correlation of SA parameters with specific behavioral traits indicates the influence of pleiotropic genes affecting both parameters of oxycodone SA and specific behavioral traits modulated by sex, Significant 3-way correlations of PR breakpoint x NOI-distance to center, PR breakpoint x total oxycodone intake, and total oxycodone intake x NOI-distance to center strongly suggest that one set of pleiotropic genes may underlie these correlations in males. It is most likely that these genes directly modulate oxycodone and novelty seeking behavior, because behavioral tests were conducted in drug naive rats. Novelty-seeking and motivation to take oxycodone may interact and regulate oxycodone intake depending on the strain-specific expression of these pleiotropic genes in males.

In summary, active licks and oxycodone intake in multiple phases of our experimental protocol were significantly greater in females of most strains. In both sexes, 16-h vs 4-h intake increased and 4-h intake predicted 16-h intake in both sexes. A subset of strains in each sex augmented their intake during the first 4-h of 16-h sessions, demonstrating genetic modulation of reward efficacy. The genome-wide search for quantitative trait loci (QTLs) based on mapping these strongly heritable SA variables and behavioral traits is likely to yield positive results. Overall, these studies demonstrate the strong strain-dependent inheritance of multiple oxycodone SA parameters and their correlations with independent measures of anxiety and novelty in established behavioral tests (i.e., EPM, OFT, NOI). The identification of these stable, within-strain behavioral phenotypes for oxycodone SA and related behaviors across a large panel of inbred rats will facilitate the discovery of genes mediating vulnerability to oxycodone and opioid misuse.

## Supporting information

Fig S1 Correlation between oxycodone and behavioral traits in females. Significant correlations (Pearson; p  <  0.05) identified between a single behavioral trait (listed on top X axis for OFT, NOI, and EPM tests) and an oxycodone self-administration parameter (Y axis). Correlation values are shown as colored dots, according to the color spectrum in the column (right, Y axis). (DOCX)

Fig S2 Correlation between oxycodone and behavioral traits in males Significant correlations (Pearson; p < 0.05) identified between a single behavioral trait (listed on top X axis for OFT, NOI, and EPM tests) and an oxycodone self-administration parameter (Y axis). Correlation values are shown as colored dots, according to the color spectrum in the column (right, Y axis). (DOCX)

Data SI 3 Elevated plus maze. (CSV)

Data SI 4 Novel object. (CSV)

Data SI 5 Open field. (CSV)

Data SI 6 Oxycodone self-administration. (CSV)

## References

[1] NSDUH. National survey on drug use and health. 2018 [cited 5 May 2018]. Available from: https://nsduhweb.rti.org/respweb/homepage.cfm

[2] National Center for Health Statistics. National vital statistics reports: from the centers for disease control and prevention, national center for health statistics, national vital statistics system. National Center for Health Statistics; 2007.

[3] Compton WM, Volkow ND. Improving outcomes for persons with opioid use disorders: Buprenorphine implants to improve adherence and access to care. JAMA. 2016;316(3):277–9. doi: 10.1001/jama.2016.8897 27434440

[4] Cerdá M, Santaella J, Marshall B, Kim J, Martins S. Nonmedical prescription opioid use in childhood and early adolescence predicts transitions to heroin use in young adulthood: A national study. J Pediatrics. 2015;167(5):605–12.e1-2. 10.1016/j.jpeds.2015.04.071 PMC4714948 26054942

[5] Enga RM, Jackson A, Damaj MI, Beardsley PM. Oxycodone physical dependence and its oral self-administration in C57BL/6J mice. Eur J Pharmacol. 2016;789:75–80. doi: 10.1016/j.ejphar.2016.07.006 27393461 PMC5824624

[6] Jimenez SM, Healy AF, Coelho MA, Brown CN, Kippin TE, Szumlinski KK. Variability in prescription opioid intake and reinforcement amongst 129 substrains. Genes Brain Behav. 2017;16(7):709–24. doi: 10.1111/gbb.12393 28523735

[7] Shaham Y. Immobilization stress-induced oral opioid self-administration and withdrawal in rats: role of conditioning factors and the effect of stress on “relapse” to opioid drugs. Psychopharmacology (Berl). 1993;111(4):477–85. doi: 10.1007/BF02253539 7870990

[8] Sharp BM, Fan X, Redei EE, Mulligan MK, Chen H. Sex and heredity are determinants of drug intake in a novel model of rat oral oxycodone self-administration. Genes Brain Behav. 2021;20(8):e12770. doi: 10.1111/gbb.12770 34459088 PMC8815756

[9] Edwards S, Koob GF. Escalation of drug self-administration as a hallmark of persistent addiction liability. Behav Pharmacol. 2013;24(5–6):356–62. doi: 10.1097/FBP.0b013e3283644d15 23839030 PMC3866817

[10] Tsuang MT, Lyons MJ, Meyer JM, Doyle T, Eisen SA, Goldberg J, et al. Co-occurrence of abuse of different drugs in men: the role of drug-specific and shared vulnerabilities. Arch Gen Psychiatry. 1998;55(11):967–72. doi: 10.1001/archpsyc.55.11.967 9819064

[11] Kendler KS, Jacobson KC, Prescott CA, Neale MC. Specificity of genetic and environmental risk factors for use and abuse/dependence of cannabis, cocaine, hallucinogens, sedatives, stimulants, and opiates in male twins. Am J Psychiatry. 2003;160(4):687–95. doi: 10.1176/appi.ajp.160.4.687 12668357

[12] Mavrikaki M, Pravetoni M, Page S, Potter D, Chartoff E. Oxycodone self-administration in male and female rats. Psychopharmacology (Berl). 2017;234(6):977–87. doi: 10.1007/s00213-017-4536-6 28127624 PMC7250466

[13] Tabakoff B, Smith H, Vanderlinden LA, Hoffman PL, Saba LM. Networking in biology: the hybrid rat diversity panel. Methods Mol Biol. 2019;2018:213–31. doi: 10.1007/978-1-4939-9581-3_10 31228159

[14] de Jong TV, Pan Y, Rastas P, Munro D, Tutaj M, Akil H, et al. A revamped rat reference genome improves the discovery of genetic diversity in laboratory rats. Cell Genom. 2024;4(4):100527. doi: 10.1016/j.xgen.2024.100527 38537634 PMC11019364

[15] Busto U, Sellers EM. Pharmacokinetic determinants of drug abuse and dependence. A conceptual perspective. Clin Pharmacokinet. 1986;11(2):144–53. doi: 10.2165/00003088-198611020-00004 3514044

[16] Nutt DJ, Nestor LJ. Drug pharmacokinetics and abuse liability. Oxford University Press; 2018.

[17] Doyle MR, Martinez AR, Qiao R, Dirik S, Di Ottavio F, Pascasio G, et al. Strain and sex-related behavioral variability of oxycodone dependence in rats. Neuropharmacology. 2023;237:109635. doi: 10.1016/j.neuropharm.2023.109635 37327971 PMC10353778

[18] Nieminen TH, Hagelberg NM, Saari TI, Pertovaara A, Neuvonen M, Laine K, et al. Rifampin greatly reduces the plasma concentrations of intravenous and oral oxycodone. Anesthesiology. 2009;110(6):1371–8. doi: 10.1097/ALN.0b013e31819faa54 19417618

[19] Gunturkun MH, Wang T, Chitre AS, Garcia Martinez A, Holl K, St Pierre C, et al. Genome-wide association study on three behaviors tested in an open field in heterogeneous stock rats identifies multiple loci implicated in psychiatric disorders. Front Psychiatry. 2022;13:790566. doi: 10.3389/fpsyt.2022.790566 35237186 PMC8882588

[20] Wang T, Han W, Wang B, Jiang Q, Solberg-Woods LC, Palmer AA, et al. Propensity for social interaction predicts nicotine-reinforced behaviors in outbred rats. Genes Brain Behav. 2014;13(2):202–12. doi: 10.1111/gbb.12112 24289793 PMC3934210

[21] Richardson NR, Roberts DC. Progressive ratio schedules in drug self-administration studies in rats: a method to evaluate reinforcing efficacy. J Neurosci Methods. 1996;66(1):1–11. doi: 10.1016/0165-0270(95)00153-0 8794935

[22] Hegmann JP, Possidente B. Estimating genetic correlations from inbred strains. Behav Genet. 1981;11(2):103–14. doi: 10.1007/BF01065621 7271677

[23] Mogil JS, Wilson SG, Bon K, Lee SE, Chung K, Raber P, et al. Heritability of nociception I: responses of 11 inbred mouse strains on 12 measures of nociception. Pain. 1999;80(1–2):67–82. doi: 10.1016/s0304-3959(98)00197-3 10204719

[24] Berrettini W. A brief review of the genetics and pharmacogenetics of opioid use disorders. Dialogues Clin Neurosci. 2017;19(3):229–36. doi: 10.31887/DCNS.2017.19.3/wberrettini 29302220 PMC5741106

[25] Deroche-Gamonet V, Belin D, Piazza PV. Evidence for addiction-like behavior in the rat. Science. 2004;305(5686):1014–7. doi: 10.1126/science.1099020 15310906

[26] Chan S, Edwards SR, Wyse BD, Smith MT. Sex differences in the pharmacokinetics, oxidative metabolism and oral bioavailability of oxycodone in the Sprague-Dawley rat. Clin Exp Pharmacol Physiol. 2008;35(3):295–302. doi: 10.1111/j.1440-1681.2007.04821.x 17973932

[27] Karami M, Zarrindast MR. Morphine sex-dependently induced place conditioning in adult Wistar rats. Eur J Pharmacol. 2008;582(1–3):78–87. doi: 10.1016/j.ejphar.2007.12.010 18191832

[28] Cicero TJ, Aylward SC, Meyer ER. Gender differences in the intravenous self-administration of mu opiate agonists. Pharmacol Biochem Behav. 2003;74(3):541–9. doi: 10.1016/s0091-3057(02)01039-0 12543217

[29] Cicero T, Ennis T, Ogden J, Meyer E. Gender differences in the reinforcing properties of morphine. Pharmacol Biochem Behav. 2000;65:91–6. 10638641 10.1016/s0091-3057(99)00174-4

[30] Yoest KE, Quigley JA, Becker JB. Rapid effects of ovarian hormones in dorsal striatum and nucleus accumbens. Hormones Behav. 2018;104:119–29. 10.1016/j.yhbeh.2018.04.002 PMC6197937 29626485

[31] Kalivas PW, Volkow ND. The neural basis of addiction: a pathology of motivation and choice. Am J Psychiatry. 2005;162(8):1403–13. doi: 10.1176/appi.ajp.162.8.1403 16055761

[32] Calipari ES, Juarez B, Morel C, Walker DM, Cahill ME, Ribeiro E, et al. Dopaminergic dynamics underlying sex-specific cocaine reward. Nat Commun. 2017;8:13877. doi: 10.1038/ncomms13877 28072417 PMC5234081

[33] Shams WM, Sanio C, Quinlan MG, Brake WG. 17β-Estradiol infusions into the dorsal striatum rapidly increase dorsal striatal dopamine release in vivo. Neuroscience. 2016;330:162–70. doi: 10.1016/j.neuroscience.2016.05.049 27256507

[34] Barbosa-Leiker C, Campbell ANC, McHugh RK, Guille C, Greenfield SF. Opioid Use Disorder in Women and the Implications for Treatment. Psychiatr Res Clin Pract. 2021;3(1):3–11. doi: 10.1176/appi.prcp.20190051 34870109 PMC8639162

[35] Office on Women’s Health. Final report: Opioid use, misuse, and overdose in women; 2017.

[36] Jamison RN, Butler SF, Budman SH, Edwards RR, Wasan AD. Gender differences in risk factors for aberrant prescription opioid use. J Pain. 2010;11(4):312–20. doi: 10.1016/j.jpain.2009.07.016 19944648 PMC2847642

[37] Back SE, Lawson KM, Singleton LM, Brady KT. Characteristics and correlates of men and women with prescription opioid dependence. Addict Behav. 2011;36(8):829–34. doi: 10.1016/j.addbeh.2011.03.013 21514061 PMC3164361

