# Supplementary figures and images for: Inbred rat heredity and sex affect oral oxycodone self-administration and augmented intake in long sessions: correlations with anxiety and novelty-seeking
Source: PLoS One. 2025 Mar 10;20(3):e0314777. doi: 10.1371/journal.pone.0314777 (PMC11892884; doi:10.1371/journal.pone.0314777)

**Figure S1. Correlation between oxycodone and behavioral traits in females**

**
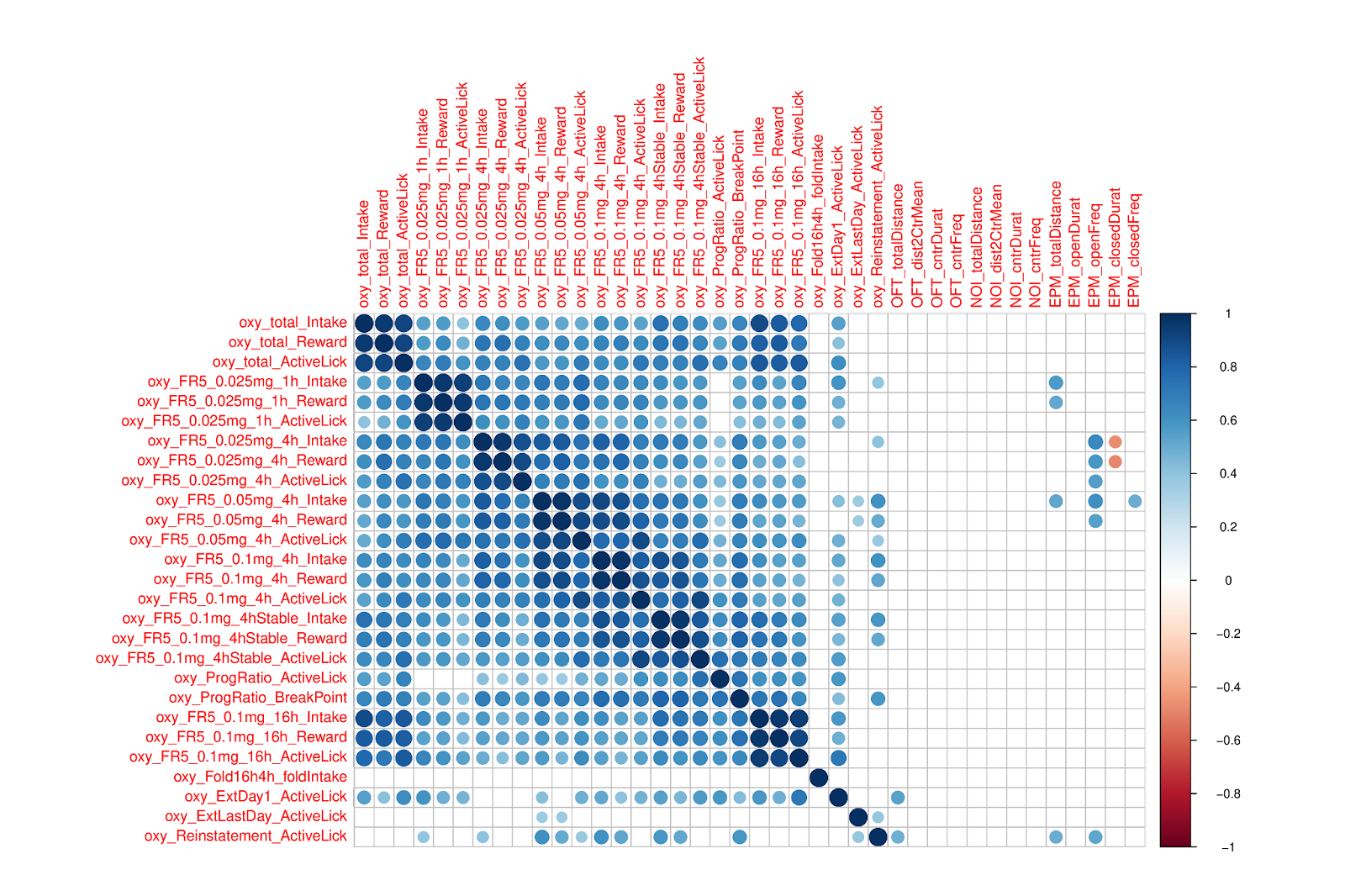
**

Supplement: Fig S1 — (DOCX) [file pone.0314777.s001.docx]

**Figure S2. Correlation between oxycodone and behavioral traits in males**

**
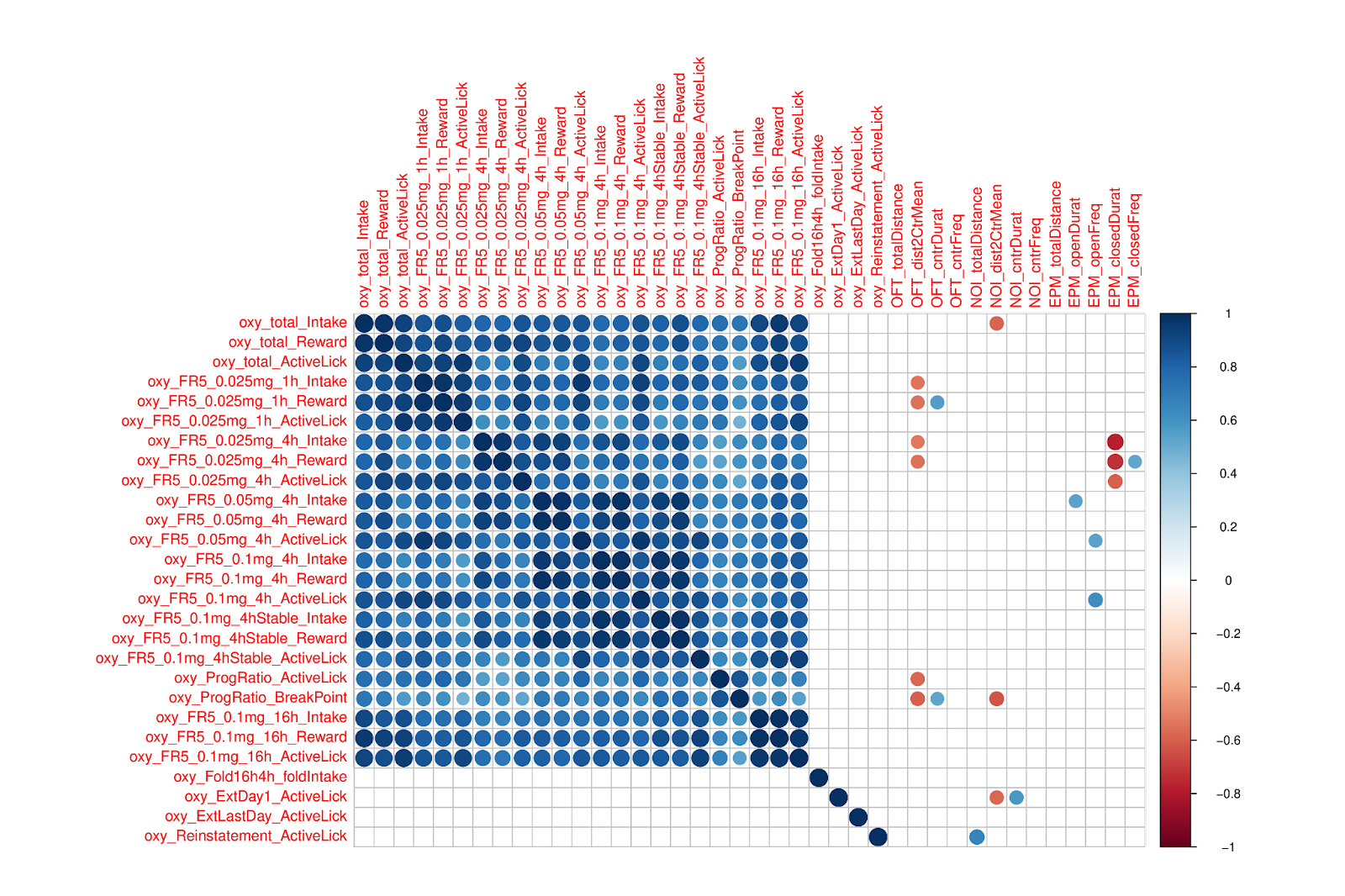
**

Supplement: Fig S2 — (DOCX) [file pone.0314777.s002.docx]
